# Seasonal variations in group leaf characteristics in species with red young leaves

**DOI:** 10.1038/s41598-019-52753-x

**Published:** 2019-11-11

**Authors:** Tai-Jie Zhang, Xing-Shan Tian, Xiao-Tao Liu, Xuan-Dong Huang, Chang-Lian Peng

**Affiliations:** 1 Guangdong Provincial Key Laboratory of High Technology for Plant Protection, Institute of Plant Protection, Guangdong Academy of Agricultural Sciences, Guangzhou, 510640 PR China; 2 Guangdong Provincial Key Laboratory of Biotechnology for Plant Development, Guangzhou Key Laboratory of Subtropical Biodiversity and Biomonitoring, School of Life Sciences, South China Normal University, Guangzhou, 510631 PR China

**Keywords:** Plant ecology, Ecophysiology, Forest ecology

## Abstract

The leaves of many plants are red during particular stages of their lives, but the adaptive significance of leaf colouration is not yet clearly understood. In order to reveal whether anthocyanins play a similar role (i.e. antioxidants) in different seasonal contexts, this study investigated species with red young leaves in the subtropical forest of Dinghushan biosphere reserve (South China) during summer and winter and compared group leaf characteristics between the two seasons. Of 62 total species, 33 exhibited red young leaves in summer only, 6 in winter only, and 23 in both seasons. The anthocyanins extracted from most of these species had an absorption peak at ~530 nm. Frequency distribution analysis showed that the species containing anthocyanins at levels ranging from 0.02 to 0.04 μmol cm^−2^ occurred most frequently in summer or winter. Based on conditional grouping of the species, no significant variation was observed in the average anthocyanin contents and antioxidant abilities between summer and winter; the flavonoid content in summer was 2-fold that in winter, whereas the anthocyanin:flavonoid ratio in summer was only half that in winter. Moreover, a positive correlation between anthocyanins and flavonoids was found in summer. Therefore, it is less likely for anthocyanins to serve as antioxidants in summer than winter, because such a function in summer leaves is readily replaced by other flavonoids.

## Introduction

In tropical and subtropical evergreen broad-leaved forests, the conspicuous red to purple pigmentation of young leaves due to the presence of anthocyanins can be observed during all seasons, including winter^1^. This developmental pattern is extremely common, and according to previous investigations conducted in tropical forests, 7% to 62% of the total vascular flora in a single site exhibited red pigmentation in the young leaves^2^. Red pigmentation is present in a variety of unrelated plant families and can be found in the vacuoles of different leaf cell layers or features, including the adaxial/abaxial epidermis, palisade mesophyll, spongy mesophyll, bundle sheath cells and trichomes^3,4^. Red pigmentation is influenced by one or more stress conditions, such as strong light^5^, UV-B radiation^6^, low temperatures^7^, low pH^8^, drought^9^, nutrient deficiencies^10^, pathogen infections and herbivory^11^. In nature, anthocyanins are present mainly in the form of hybrid heterosides, and their compositions can change in response to different stresses even in the same species^12^.

The biological functions of anthocyanins in leaves are as varied as their cellular location and environmental stimuli. Current research has shown that the anthocyanins that accumulate in leaves can act as ROS scavengers^13^, light screens^14^, metal chelators^15^, and antimicrobial agents^16^. Anthocyanins may also signal the presence of chemical defences^17^ or function in crypsis to herbivores^18^. Recently, an increasing number of documents showed that anthocyanins participate in photoprotection by screening the excessive light energy. In general, anthocyanins absorb visible light with a peak in the 500–550 nm waveband^19^. Thus, anthocyanins can serve a photoprotective function by absorbing excessive blue-green light that could otherwise be absorbed by chloroplasts in the subjacent mesophyll^20^. The light energy absorbed by anthocyanins is eventually dissipated into heat through a series of ultra-fast excited-state processes^21^. In addition to absorbing blue-green light, anthocyanins can also scavenge free radicals. However, as anthocyanins constitute only a minor portion of the total antioxidant pool in leaves, whether they protect plants from oxidative stress by acting as scavengers of free radicals remains a subject of debate. Anthocyanins belong to the group of compounds known as flavonoids, and most flavonoids are powerful antioxidants because of their strong capacity to donate electrons or hydrogen atoms^22^. Therefore, analysing the relationships between the accumulation of anthocyanins, flavonoids and antioxidants in specific groups of species and in a specific site may increase our understanding of whether it is necessary for anthocyanins to function as antioxidants.

Dinghushan biosphere reserve (DHSBR) is the first natural reserve in China and is located near the Tropic of Cancer. Two-thirds of the global area near this latitude consists of deserts and semideserts. However, the DHSBR is covered by a subtropical evergreen broad-leaved forest, and the climax community in the area is more than 400 years old^23^. Because of its unique geographical location and the presence of native vegetation with little human interference, the DHSBR has become one of the most valuable areas for scientific research. Like many other evergreen broad-leaved forest, the subtropical forest has abundant plants with coloured leaves. To better understand whether anthocyanins play an antioxidant role in the plants in DHSBR area, species with red young leaves during summer and winter were investigated, and the absorption spectra of anthocyanins, the relationships among anthocyanin contents, flavonoid contents and antioxidant abilities in different groups of species were explored during the two contrasting seasons.

## Results

### Species with red young leaves in the DHSBR in summer and winter

The DHSBR forest was rich in plant species with red young leaves, most of which were woody species (Table 1; Fig. S1 available as Additional Information). A total of 62 different species were observed with red (with anthocyanin) young leaves in the two field investigations, and species examined consisted of 38 trees, 11 shrubs, 12 vines and 1 herb. Among the species, *Lindera chunii* and *Macaranga sampsonii* are endemic plants in the DHSBR. The number of species (56) with red young leaves was greater in summer than in winter (29). There were 33 and 6 species with red young leaves only in summer or winter, respectively. Twenty-three species had red young leaves in both seasons, of which 5 species (*Acmena acuminatissima*, *Ficus fistulosa, Machilus chinensis, Blastus cochinchinensis, Eriocarpous Glochidion* and *Rourea microphylla*) were visibly redder in winter than in summer, and 2 species (*Craibiodendron kwangtungense* and *Erycibe schmidtii*) were the opposite. We noted that the young leaves of some of the species were not really red; for example, those of *Xanthophyllum hainanense* and *Smilax china* were blue and purple-black, respectively. In addition to showing temporal diversity, there was also diversity in the distribution of anthocyanin pigments in the tissues of the plants in the DHSBR. For most of the species, there was pigmentation on both the adaxial and abaxial sides of the young leaves. However, for a few species, there was pigmentation only on the adaxial side (such as *Eurya groffii* and *E. Glochidion*), on the abaxial side (such as *Melastoma sanguineum*), or in trichome (such as *Castanopsis fissa* and *Schefflera octophylla*).

**Table 1:** Species with red young leaves in the Dinghushan biosphere reserve.

Species	Abbr.	Reddening season	Reddening Site^a^	Growth form	Family
*Acmena acuminatissima*	Aac	Sum/Win	Ad/Ab	Tree	Myrtaceae
*Aporosa dioica*	Adi	Sum/Win	Ad/Ab	Tree	Euphorbiaceae
*Brassaiopsis glomerulata*	Bgl	Sum	Ad/Ab	Tree	Araliaceae
*Calophyllum membranaceum*	Cme	Win	Ad/Ab	Tree	Guttiferae
*Camellia euryoides*	Ceu	Sum	Ad/Ab	Tree	Theaceae
*Castanopsis fissa*	Cfi	Sum/Win	T	Tree	Fagaceae
*Castanopsis chinensis*	Cch	Sum	Ad/Ab	Tree	Fagaceae
*Cinnamomum cassia*	Cca	Win	Ad/Ab	Tree	Lauraceae
*Craibiodendron kwangtungense*	Ckw	Sum/Win	Ad/Ab	Tree	Ericaceae
*Cratoxylum cochinchinense*	Cco	Sum	Ad/Ab	Tree	Guttiferae
*Cryptocarya concinna*	Ccn	Sum	Ad/Ab	Tree	Lauraceae
*Cryptocarya chinensis*	Cci	Win	Ad/Ab	Tree	Lauraceae
*Diospyros strigosa*	Dst	Sum/Win	Ad/Ab	Tree	Ebenaceae
*Engelhardtia roxburghiana*	Ero	Sum/Win	Ad/Ab	Tree	Juglandaceae
*Eurya groffii*	Egr	Win	Ad	Tree	Theaceae
*Ficus esquiroliana*	Fes	Win	Ad/Ab	Tree	Moraceae
*Ficus fistulosa*	Ffi	Sum/Win	Ad/Ab	Tree	Moraceae
*Garcinia oblongifolia*	Gob	Sum/Win	Ad/Ab	Tree	Guttiferae
*Homalium cochinchinense*	Hco	Sum	Ad/Ab	Tree	Flacourtiaceae
*Ilex pubescens*	Hpu	Sum	Ad/Ab	Tree	Aquifoliaceae
*Lindera chunii*	Lch	Sum	Ad/Ab	Tree	Lauraceae
*Macaranga sampsonii*	Msa	Sum/Win	Ad/Ab	Tree	Euphorbiaceae
*Machilus chinensis*	Mch	Sum/Win	Ad/Ab	Tree	Lauraceae
*Machilus pingii*	Mpi	Sum	Ad/Ab	Tree	Lauraceae
*Machilus velutina*	Mve	Sum	Ad/Ab	Tree	Lauraceae
*Memecylon nigrescens*	Mni	Sum/Win	Ad/Ab	Tree	Melastomataceae
*Microcos paniculata*	Mpa	Sum	Ad/Ab	Tree	Tiliaceae
*Olea europaea*	Oeu	Sum	Ad/Ab	Tree	Burseraceae
*Rhaphiolepis indica*	Rin	Sum/Win	Ad/Ab	Tree	Rosaceae
*Rhus chinensis*	Rcn	Sum	Ad/Ab	Tree	Anacardiaceae
*Sapium discolor*	Sdi	Sum	Ad/Ab	Tree	Euphorbiaceae
*Sarcosperma pedunculatum*	Spe	Sum	Ad/Ab	Tree	Sapotaceae
*Schefflera octophylla*	Sot	Sum/Win	T	Tree	Araliaceae
*Schima superba*	Ssu	Sum	Ad/Ab	Tree	Theaceae
*Syzygium jambos*	Sja	Sum/Win	Ad/Ab	Tree	Myrtaceae
*Syzygium rehderianum*	Sre	Sum/Win	Ad/Ab	Tree	Myrtaceae
*Toxicodendron vernicifluum*	Ter	Sum	Ad/Ab	Tree	Anacardiaceae
*Xanthophyllum hainanense*	Xha	Sum/Win	Ad/Ab	Tree	Xanthophyllaceae
*Alchornea trewioides*	Atr	Sum	Ad/Ab	Shrub	Euphorbiaceae
*Blastus cochinchinensis*	Bco	Sum/Win	Ad/Ab	Shrub	Melastomataceae
*Dendropanax proteus*	Dpr	Sum	Ad/Ab	Shrub	Araliaceae
*Desmos chinensis*	Dch	Sum/Win	Ad/Ab	Shrub	Annonaceae
*Eriocarpous glochidion*	Egl	Sum/Win	Ad	Shrub	Euphorbiaceae
*Ixora chinensis*	Ich	Sum/Win	Ad/Ab	Shrub	Rubiaceae
*Marsdenia tinctoria*	Mti	Sum	Ad/Ab	Shrub	Asclepiadaceae
*Melastoma sanguineum*	Msn	Sum/Win	Ab	Shrub	Melastomataceae
*Rubus alceaefolius*	Ral	Sum	Ad/Ab	Shrub	Rosaceae
*Taxillus chinensis*	Tch	Win	Ad/Ab	Shrub	Loranthaceae
*Uvaria macrophylla*	Uma	Sum	Ad/Ab	Shrub	Annonaceae
*Bauhinia championi*	Bch	Sum	Ad/Ab	Vine	Fabaceae
*Byttneria aspera*	Bas	Sum	Ad/Ab	Vine	Sterculiaceae
*Caesalpinia crista*	Ccr	Sum	Ad/Ab	Vine	Caesalpiniaceae
*Capparis acutifolia*	Cac	Sum	Ad/Ab	Vine	Capparidaceae
*Erycibe schmidtii*	Esc	Sum/Win	Ad/Ab	Vine	Convolvulaceae
*Fissistigma glaucescens*	Fgl	Sum	Ad/Ab	Vine	Annonaceae
*Millettia dielsiana*	Mdi	Sum	Ad/Ab	Vine	Fabaceae
*Rourea microphylla*	Rmi	Sum/Win	Ad/Ab	Vine	Connaraceae
*Sabia limoniacea*	Sli	Sum	Ad/Ab	Vine	Sabiaceae
*Smilax china*	Sch	Sum	Ad/Ab	Vine	Liliaceae
*Tetracera asiatica*	Tas	Sum	Ad/Ab	Vine	Dilleniaceae
*Uncaria rhynchophylla*	Urh	Sum	Ad/Ab	Vine	Rubiaceae
*Pteris vittata*	Pvi	Sum	Ad/Ab	Herb	Pteridaceae

Notes: ^a^Reddening site is judged by the naked eyes. Sum is summer; Win, winter; Ad, adaxial side; Ab, abaxial side; T, trichome.

### Absorption spectra and anthocyanin contents of the different species

**Figure 1:**
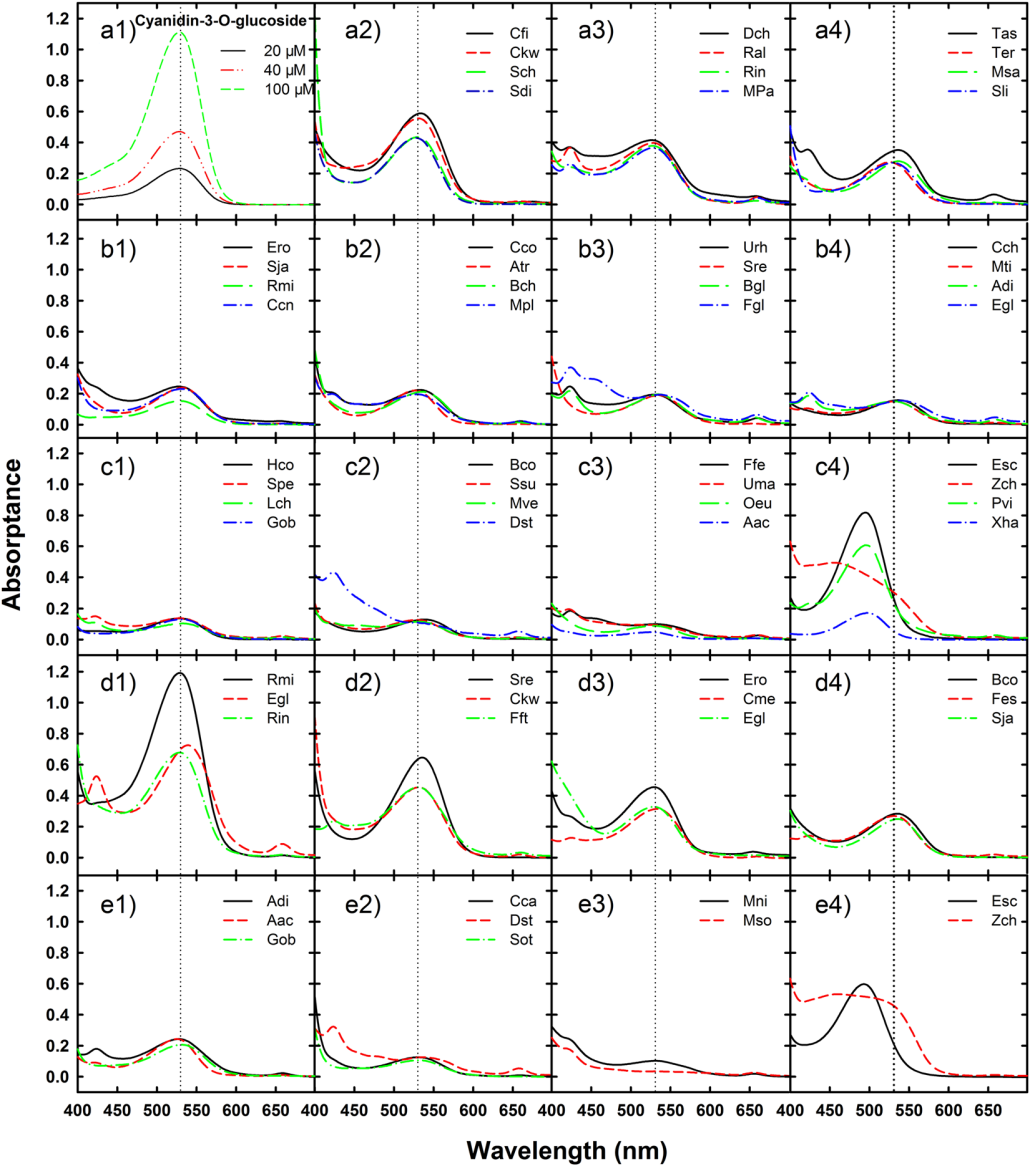
Absorption spectra of the anthocyanin extracts from the red young leaves of 44 and 22 species in the Dinghushan biosphere reserve in summer (panels a2-c4) and winter (panels d1-e4), respectively. Panel a1 is the anthocyanin standard cyanidin-3-O-glucoside. Vertical dotted lines indicate a wavelength at 530 nm. According to visually-similar absorption peaks and the absorbance at 530 nm from high to low, every 4 species formed a group in summer and every 2 or 3 species formed a group in winter.

**Figure 2:**
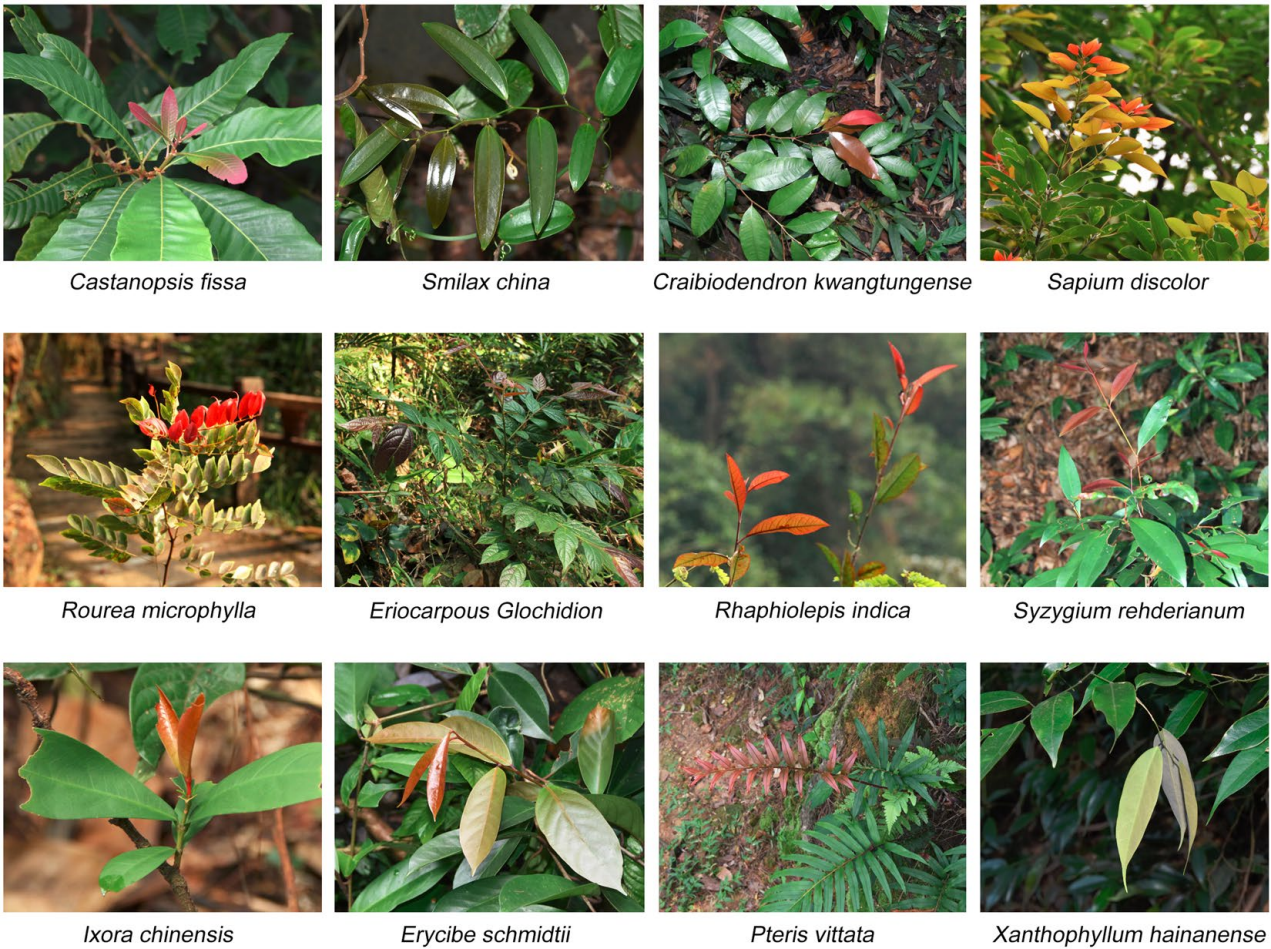
Some outstanding species in the Dinghushan biosphere reserve. The upper and middle 4 panels were the top 4 species with the highest accumulated anthocyanins in their young leaves in summer and winter, respectively. The species in lower panels exhibited absorption spectra that are different from most other plants with red leaves. (Photo by T.-J. Zhang).

Leaf samples of 44 species and 22 species were collected in the DHSBR during summer and winter, respectively, and the absorption spectra of their anthocyanin extracts are shown in Fig. 1. As seen in Fig. 1, 40 out of 44 species in summer (Fig. 1(a2–c4)) and 20 out of 22 species in winter (Fig. 1(d1–e4)) had absorption peaks at 530 ± 5 nm, which was consistent with the anthocyanin standard, cyanidin-3-O-glucoside (Fig. 1(a1)). *C. fissa*, *S. china*, *C. kwangtungense* and *Sapium discolor* accumulated the most anthocyanins in summer, and *R. microphylla*, *E. Glochidion*, *Rhaphiolepis indica* and *Syzygium rehderianum* accumulated the most in winter (Figs. 1 and 2). Unlike most species, the absorption peaks of *E. schmidtii*, *Pteris vittata* and *X. hainanense* in summer occurred at approximately 496 nm rather than 530 nm (Figs 1 and 2). Among the three species, only *E. schmidtii* was found to have young red leaves in both summer and winter, and the sample collected in winter showed an absorption spectrum that was identical to that in summer. *Ixora chinensis* was the only species that did not show an evident absorption peak in its spectrum either in summer or winter. With wavelengths ranging from 600 to 450 nm, the absorption spectra of *Ixora chinensis* showed a distinctive increasing trend.

**Figure 3:**
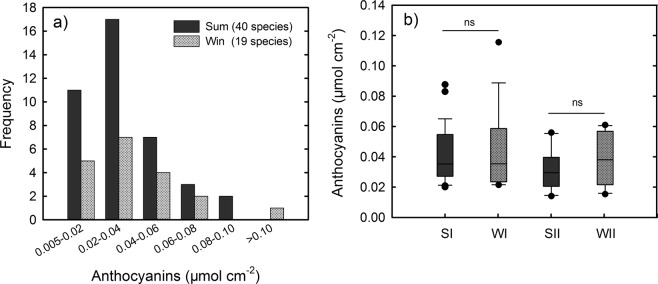
(**a**) Frequency histogram of the species whose young leaves had anthocyanin contents >0.005 μmol cm^−2^ in summer (Sum) and winter (Win). (**b**) Average anthocyanin content in the young leaves of the 4 species groups (SI, WI, SII, and WII) in the two seasons. Statistical significant differences are indicated: ns, non-significant; *P < 0.05; **P < 0.01; ***P < 0.001.

The anthocyanin content of the species with an absorption peak at approximately 530 nm was calculated using cyanidin-3-O-glucoside as the standard. The frequency distributions of 40 species in summer and 19 species in winter based on anthocyanin content are shown in Fig. 3a. The anthocyanin contents that occurred most frequently ranged from 0.02–0.04 μmol cm^−2^, with 17 species in summer and 7 species in winter. In order to facilitate the comparisons of anthocyanins and other characteristics between summer and winter, the species were classified: those in summer and winter with anthocyanin contents >0.02 μmol cm^−2^ refer as SI and WI groups, respectively; for those with red young leaves in both the two seasons, they refer as SII and WII groups when in summer and winter, respectively. The statistical results showed that there were no significant variation in the mean anthocyanin contents between the SI and WI groups or between the SII and WII groups (Fig. 3b).

### Chlorophyll (Chl) content in the different species groups

**Figure 4:**
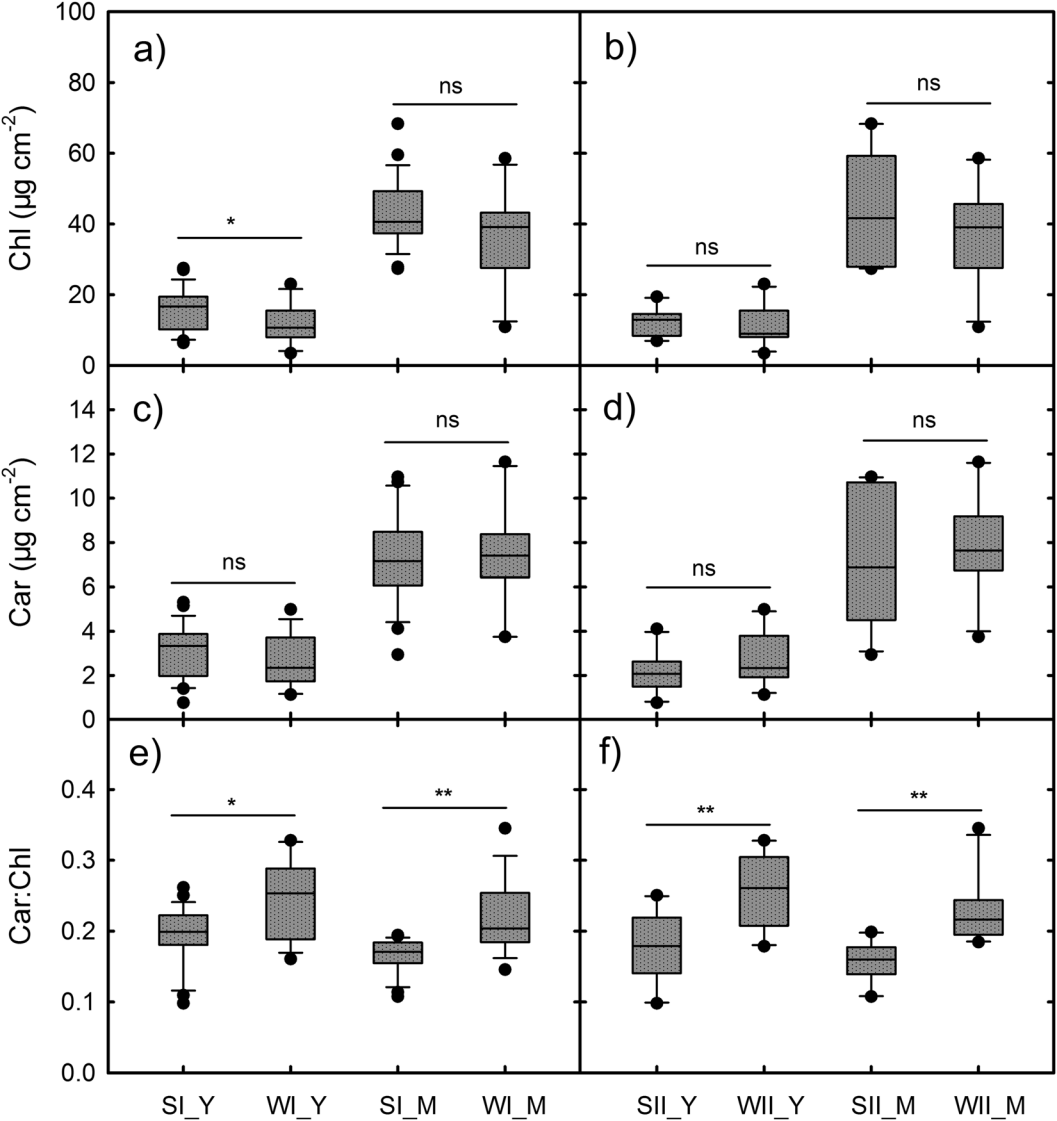
Chlorophyll and carotenoid content in the young red (Y) and mature leaves (M) of the 4 species groups (SI, WI, SII, and WII) in the two seasons. Statistical significant differences are indicated: ns, non-significant; *P < 0.05; **P < 0.01; ***P < 0.001. The x-axis labels correspond to the group leaf type, e.g., SI_Y represent young leaves in the SI group.

The mean Chl content was higher in the young leaves of the SI group than of the WI group (*P* < 0.05), while the mean Car content was not significantly different between the two groups (Fig. 4). As a result, the Car:Chl ratio in the young leaves of the SI group was significantly lower than that of the WI group (*P* < 0.05). Similar changes in Chl content, Car content and Car:Chl ratio were found in the mature leaves between the SI and WI groups and in the young and mature leaves between the SII and WII groups.

### Antioxidant abilities and flavonoid contents in the different species groups

**Figure 5:**
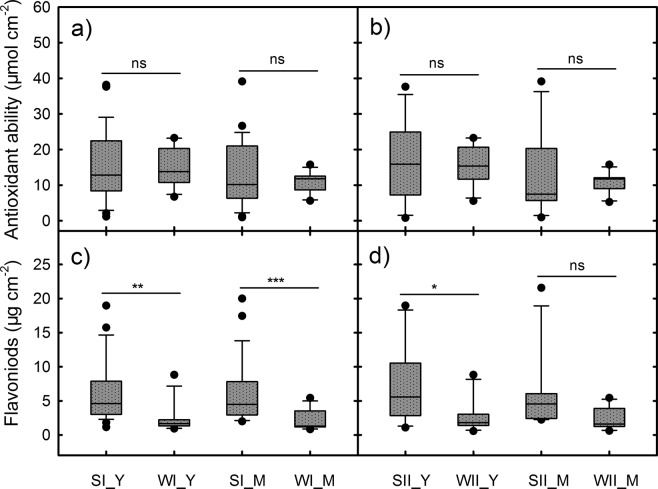
Antioxidant abilities and total flavonoid contents in the young red (Y) and mature leaves (M) of the 4 species groups (SI, WI, SII, and WII) in the two seasons. Statistical significant differences are indicated: ns, non-significant; *P < 0.05; **P < 0.01; ***P < 0.001. The x-axis labels correspond to the group leaf type, e.g., SI_Y represents young leaves in the SI group.

**Figure 6:**
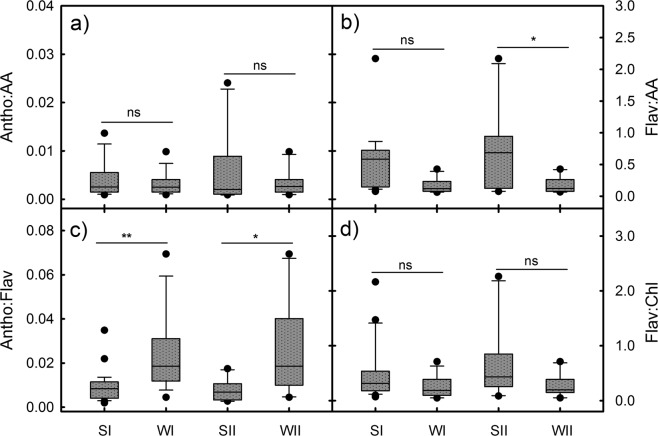
Anthocyanins:antioxidant abilities (Antho:AA), anthocyanins:flavonoids (Antho:Flav), flavonoids:antioxidant abilities (Flav:AA) and flavonoids:chlorophylls (Flav:Chl) in the young red leaves of the 4 species groups (SI, WI, SII, and WII) in the two seasons. Statistical significant differences are indicated: ns, non-significant; *P < 0.05; **P < 0.01; ***P < 0.001.

There were no significant differences in the average antioxidant abilities in the young or mature leaves between the SI and WI groups (Fig. 5a,b). In contrast, the average flavonoid content was 1-fold greater in the young or mature leaves of the SI group than in the WI group (Fig. 5c) and 1.8–2.2-fold greater in the young or mature leaves of the SII group than in the WII group (Fig. 5d). The effects of different seasons on the accumulation of flavonoids in the young leaves of the species groups were apparently different from those of the anthocyanins. The summer groups accumulated more flavonoids than the winter groups, and therefore, the differences in the flavonoids:antioxidant abilities (Flav:AA) ratios between either the SI and WI groups or the SII and WII groups were greater than those in the anthocyanins:antioxidant abilities (Antho:AA) ratios (Fig. 6a,b). Moreover, the Antho:Flav ratios in the young leaves of the SI and SII groups were only half those of the WI and WII groups (Fig. 6c). Instead, the SI and SII groups had the Flav:Chl ratios that were slightly higher than the corresponding winter groups (Fig. 6d).

### Correlations between anthocyanins and flavonoids

**Figure 7:**
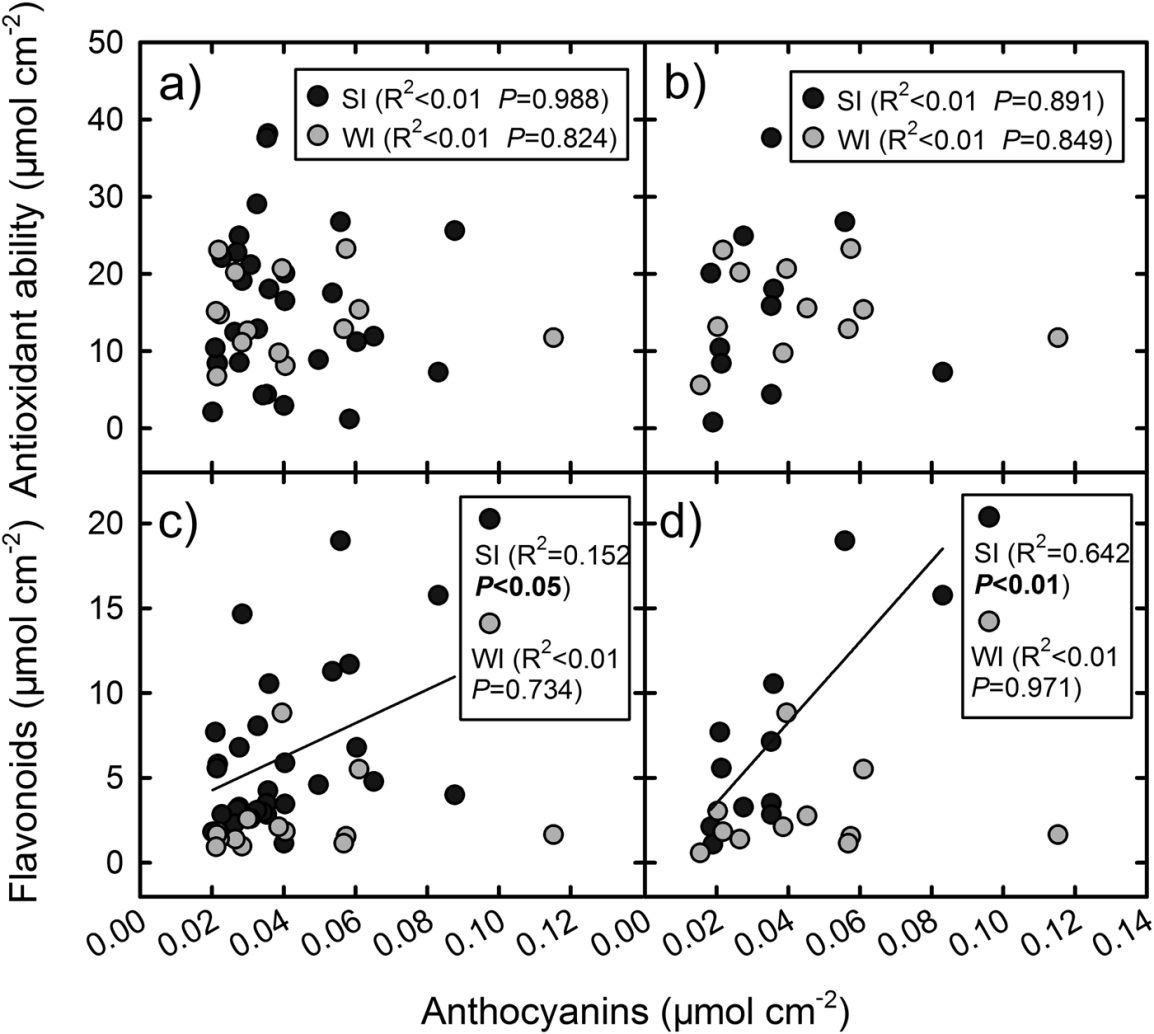
Correlations between the anthocyanin contents and the antioxidant abilities and flavonoid contents in the young leaves in the 4 species groups (SI, WI, SII, and WII) in two seasons. A significant correlation was only found in SI and SII in the two lower panels, and that the best fit lines only correspond with those data.

**Figure 8:**
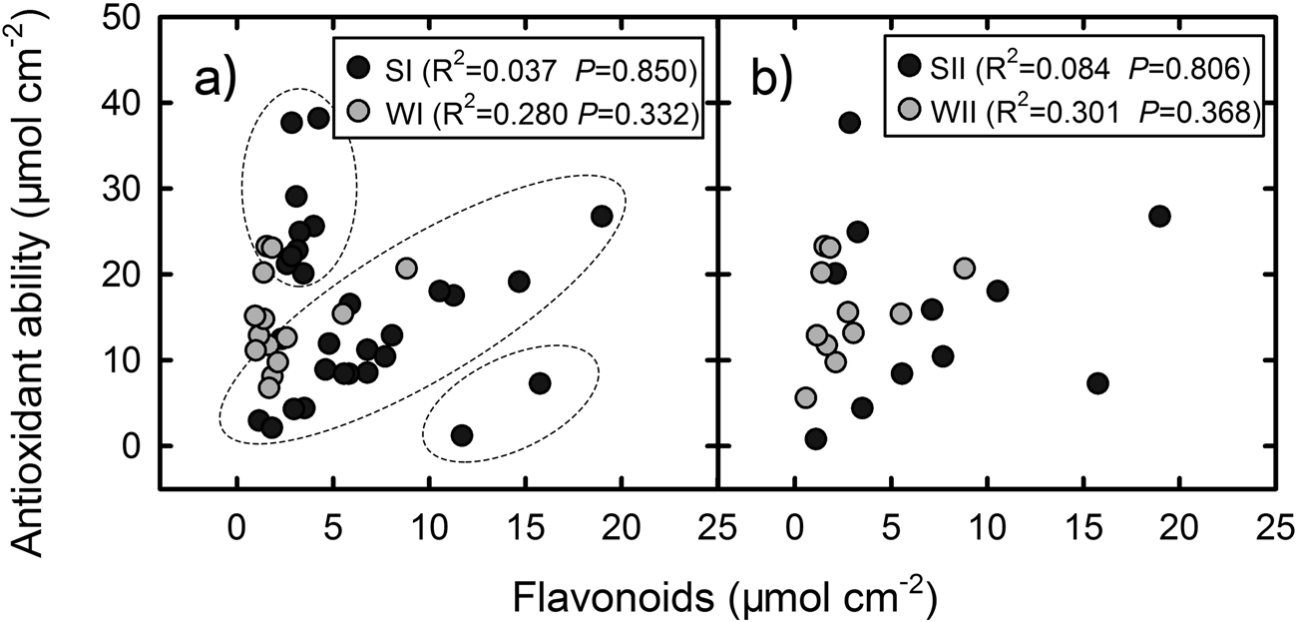
Correlations between the antioxidant abilities and flavonoid contents in the young leaves in the 4 species groups (SI, WI, SII, and WII) in two seasons.

Anthocyanins represent a portion of the flavonoid pool and antioxidant ability in the red leaves. However, there was no linear relationship between the anthocyanins and the antioxidant abilities or between the flavonoids and the antioxidant abilities in the young leaves of any of SI, WI, SII, and WII groups (Figs. 7 and 8). Anthocyanins were positively correlated with flavonoids in the SI group (*P* < 0.05) and SII group (*P* < 0.01) but not in the WI and WII groups (Fig. 7c,d). According the distribution of antioxidant abilities versus flavonoids (Fig. 8a), the species in SI group tended to be separated into three subgroups (indicated by the dotted oval). One was characterized by high antioxidant abilities and low flavonoids, including 8 species (e.g. *Syzygium jambos*, *Alchornea trewioides* and *Syzygium rehderianum*). Another included the most species (e.g. *Rubus alceaefolius*, *Rhaphiolepis indica* and *Microcos paniculata*), in which the antioxidant abilities were associated with flavonoids. The third had two species (*Craibiodendron kwangtungense* and *Desmos chinensis*), which was characterized by low antioxidant abilities and high flavonoids.

## Discussion

### Species with red young leaves

Some species in the DHSBR continuously produce new leaves all year round, other produce them only during specific seasons. This may represent different kinds of life history strategies. But the pigmentation pattern of young leaves is not directly related to any specific life history strategy, because there are always some species with young red leaves in the DHSBR all year round. Our investigations found that 62 different species produced new leaves that are red in the DHSBR during summer and winter. Since our investigations were carried out for a limited period of time and in a limited area, the result was unable to cover all plant species with young red leaves in the DHSBR. For example, a previous investigation has demonstrated that the young leaves of *Lindera communis*, *Litsea verticillata*, *Rhododendron henryi* and *Elaeocarpus chinensis* in the forest were also red^24^. So, on a conservative estimate, there may be approximately 80 species with red young leaves in the DHSBR, of which 70 are woody species. Based on the investigation of a 20 ha plot, there are 210 woody species in the DHSBR^25^. Therefore, woody species with red young leaves account for a third of the total woody species in the subtropical forest, which included nearly all the dominant species.

### Absorbance profiles of anthocyanins in the young red leaves

Absorption of the anthocyanin extracts varied in different species may due to different compositions of the pigments. For 90% of the species investigated in this study, the absorption peaks occurred at 530 ± 5 nm, which are roughly consistent with consistent with cyanidin-3-O-glucoside (Fig. 1), a common anthocyanin in pant vegetative organs. Though cyanidin-3-O-glucoside is not the only anthocyanin that absorbs at 530 nm^26^, it is a useful standard for estimation of anthocyanin contents in most of the species. To understand the exact compositions of anthocyanins in the various species, much more work is needed. Nevertheless, cyanidin derivatives (not necessarily cyanidin-3-O-glucoside) have been extensively identified in the vegetative tissues of many plants^27–29^. So, it is likely that the red pigments accumulated in the species with maximum absorption at about 530 nm consisted mainly of cyanidin derivatives. A few species showed absorption peaks at approximately 496 nm (including *Erycibe schmidtii*, *Pteris vittata* and *X. hainanense*) or did not even have an evident absorption peak (i.e., *Ixora chinensis*), suggesting that compositions of anthocyanins in these species are very different from most of other species. As the pigments with an absorption peak at ~496 nm is consistent with that of luteolinidin-5-O-glucoside^30^, they might contain lots of luteolinidin derivatives rather than cyanidin derivatives.

### Effects of seasonal variations on group leaf characteristics

Temperatures, irradiances and precipitations are different between summer and winter in the DHSBR area. In summer, growth and photosynthetic function in leaves are readily influenced by high temperatures and high irradiances. As the season changes, chilling temperatures and drought in winter become the major factors that inhibit the growth of leaves and their functions. The variations of the Chl and flavonoid contents indicate that the species in the DHSBR took different strategies to adapt summer winter stress. Reduced Chl content in the leaves of winter groups resulted in a higher a Car:Chl ratio (Fig. 4). Since winter is drier and colder than summer, desiccation and low-temperature stress in plants should be relatively higher, which would be accompanied by stomatal closure and photoinhibition of photosynthesis. As some specific carotenoids, such as xanthophylls, are involved in the mechanisms to cope with the absorption of excess light energy in photosynthetic apparatus, high Car:Chl ratio can imply that the capacity of photoprotection was enhanced in the leaves^31,32^. This may allow the species to adapt to winter stresses.

In contrary to the Car:Chl ratio, the flavonoids were dramatically higher in the summer leaves than the winter ones (Fig. 5). Flavonoids can be involved in photoprotection by acting as antioxidants or light attenuators. Thus, the upregulation of flavonoids in plants has been observed under various environmental stresses, such as high light^33–35^ and chilling temperatures^36–38^. Though flavonoids didn’t have a remarked effect on total antioxidant abilities either in the summer or winter leaves, the higher flavonoids could make a greater contribution to the antioxidant abilities in summer leaves. Since there was no difference in the antioxidant abilities between summer and winter leaves, flavonoids in the summer leaves may substitute some other antioxidants. Perhaps, flavonoids are better kind of antioxidants for the plants to adapt summer stresses. In addition, winter leaves accumulated less flavonoids is likely due to the coordination of different photoprotective mechanisms but not the inhibition of environmental conditions, because they still had the ability to produce large amounts of anthocyanins.

Unlike the changes of Chls and flavonoids, the anthocyanins tended to maintain at the same level between the summer and winter groups of species (Fig. 3). The anthocyanin concentrations observed in the two seasons may be adequate for achieving their functions in either season. In summer, the anthocyanins were positively correlated with flavonoids (Fig. 7), suggesting that the accumulation of anthocyanins can be partly attributed to the active metabolism of flavonoids. In other words, some anthocyanins in the young leaves may be “by-products” of flavonoid metabolism in summer. In winter, however, the production of anthocyanins were independent of other flavonoids, consistent with previous studies showing that the anthocyanins increased in some plants under environmental stresses without affecting the flavonoids^5,39^. The larger amount of falovanoids observed in summer may be caused by light-induced activation of enzymes for falovanoid pathway. As summer conditions are more beneficial than winter conditions for photosynthesis of plants, maybe there are also more available energy for the synthesis of flavonoids in summer. Nevertheless, as summer leaves had high flavonoids, the anthocyanins acting as scavengers of free radicals can be easily replaced by other flavonoids. In this respect, the photoprotective function afforded by anthocyanins in young leaves might be less important in summer than in winter. As summer temperatures are more favourable for insect herbivores than winter ones^40^, it may be more important for anthocyanins to play an anti-herbivory role in summer than in winter.

Though the difference of mean antioxidant abilities between the summer and winter groups of species was consistent with that of mean anthocyanin concentrations, a low correlation was observed between the anthocyanins and the antioxidant abilities. This might be partly caused by the fact that while anthocyanins are mainly located certain cells within the leaves, other antioxidative compounds are located in the whole profile of the leaf tissue. Future synchronous analyses of anthocyanins, flavonoids and antioxidant potentials in specific leaf tissues *in situ* may provide more accurate information for us to learn about the antioxidation effect of anthocyanins in different seasonal conditions. Moreover, future investigations of the leaf physiological characteristics in the different species groups, which are divided by anthocyanin distribution features (e.g. in epidermis or trichome) or antioxidant traits (e.g. high antioxidant abilities and low flavonoids or low antioxidant abilities and high flavonoids), will also provide insights into the adaptive significances of the anthocyanins accumulated in the young leaves of a variety of species in subtropical forests.

## Materials and Methods

### Investigation location and sampling

**Figure 9:**
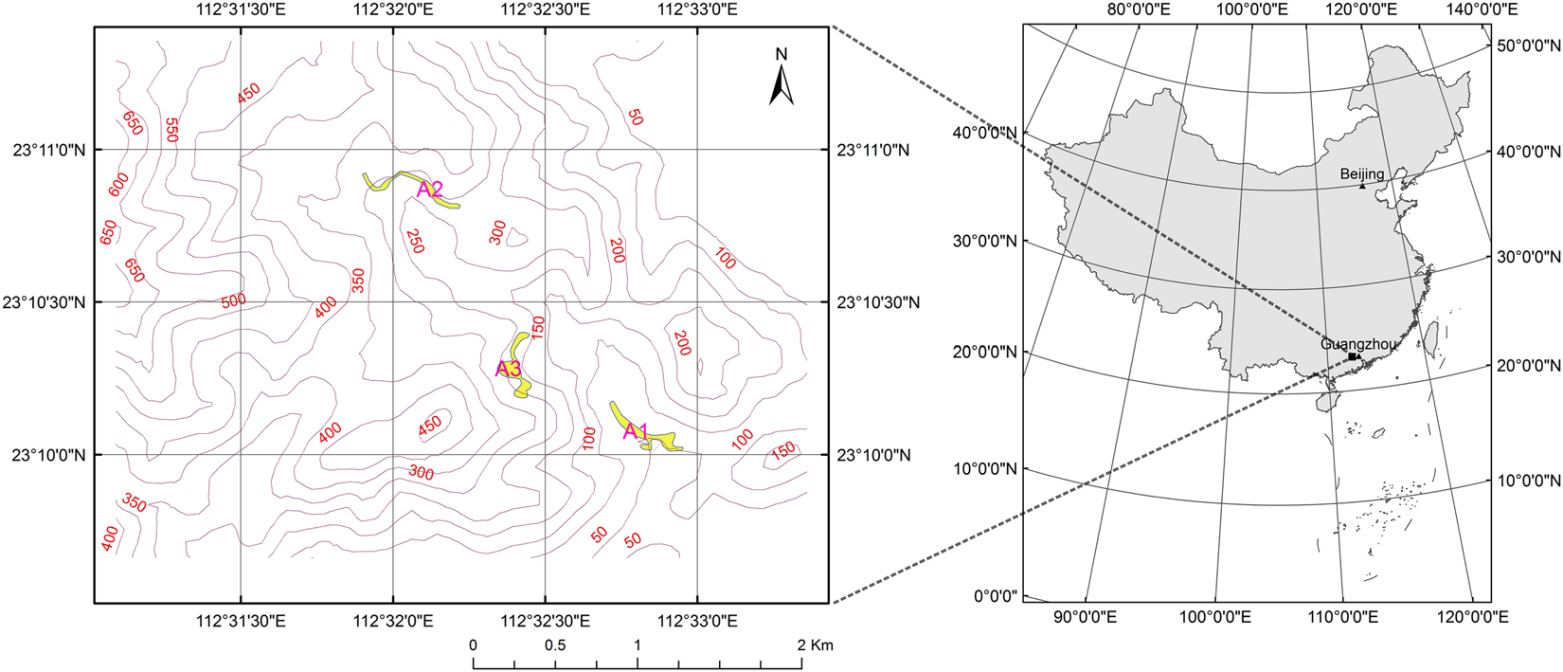
Investigation area (A1, A2 and A3) in the Dinghushan biosphere reserve. Map was drawn using ArcGIS 10.2 software (http://www.esri.com/).

The DHSBR is located at latitude 23°09′21″–23°11′30″N and longitude 112°30′39″–112°33′41″E in Zhaoqing, Guangdong Province, China, approximately 80 km west of Guangzhou. It has an area of 1154 hm^2^ and a hilly terrain, with altitudes ranging between 10 and 1000 m. The geology of this area consists of sandstone and shale bedrock formed by Devonian-aged sediments overlain with lateritic red soil. Although the DHSBR is located near the Tropic of Cancer, the area has a subtropical monsoon humid climate due to the effect of the Pacific and Indian Oceans. The mean annual temperature is 21 °C, and the temperatures in summer and winter are on average 28.0 °C and 12.0 °C, respectively. Occasionally, they can reach to 38 °C in summer and −2 °C in winter^41^. The annual rainfall ranges from 1560 to 2278 mm, with a mean of 1900 mm, of which approximately 80% occurs from April to September. The DHSBR has 1956 species of higher plants representing 877 genera from 260 families, of which 210 species of woody plants belong to 119 genera and 56 families^25^. The investigation of plants with red young leaves in the DHSBR was performed in January (winter) and August (summer) 2016 and mainly focused on 3 areas (A1–A3, Fig. 9). Investigation areas A1, A2 and A3 represented a ravine evergreen broad-leaved forest (altitude 50–90 m), a monsoon evergreen broad-leaved secondary forest (altitude 270–320 m), and a monsoon evergreen broad-leaved forest (altitude 160–210 m), respectively. In particular, A3 was located in the climax community in the DHSBR that is more than 400 years old. Plants with red young leaves were sought along the mountain paths within these areas.

To collect samples for the determination of anthocyanin and other physiological traits in the red leaves, a pair of common branch scissors or a pair of 10 m high branch scissors was used to cut down a branch of a species with red leaves. The branch was immediately placed in a plastic bucket with the cut end in water. After returning indoors, 15–20 leaf discs (10 mm in diameter for most species; 8 mm for *Rourea microphylla* due to its small leaves) were cut off from the most striking red young leaves and fully expanded mature leaves of each species, packed in tinfoil (5 leaf discs per package), and stored in liquid nitrogen until further processing. Leaf samples were collected from 2–3 individuals of each species with red leaves.

### Determination of anthocyanin content

Anthocyanins were extracted from the red leaves in methanol containing 1% HCl. Four leaf discs were immersed in 4 mL of the HCl methanol solution in a 15 mL test tube with screw caps and placed at 4 °C in the dark for approximately 24 h until the leaf colour was completely bleached. Both anthocyanins and damaged chlorophylls (Chls) were dissolved in the extract, which made the extract appear cloudy. To separate anthocyanin from the extract, 4 mL of chloroform and 2 mL of distilled water were added and mixed. After standing for 3 minutes, the anthocyanins and Chls were dissolved in the upper water-methanol phase and the lower chloroform phase, respectively. The volume of the water-methanol phase was measured and recorded. Then, a 3 mL aliquot of the upper anthocyanin extract was transferred to a cuvette, and the absorption spectra were scanned at 1 nm wavelength intervals from 400 to 700 nm using a UV-Vis 2450 spectrophotometer (Shimadzu, Japan). The absorption peaks of the anthocyanin extract for most species occurred at approximately 530 nm, which is consistent with cyanidin-3-O-glucoside. Therefore, the anthocyanin content in these species was calculated using the absorbance at 530 nm, and a calibration curve was constructed with 5–100 μmol L^−1^ of cyanidin-3-O-glucoside (Sigma) and expressed on an area basis as μmol cm^−2^. A few species, such as *Erycibe schmidtii*, *Pteris vittata* and *Xanthophyllum hainanense*, had an absorption peak at 496 nm rather than 530 nm. The anthocyanin contents of these species were not calculated due to a lack of standards.

### Determination of the chlorophyll (Chl) content

Chls were extracted in 80% acetone. For every sample, 3 leaf discs were immersed in 4 mL of 80% acetone in a 10 mL test tube, and the screw cap was then tightened. The samples were placed at 4 °C in the dark and shaken at 8 h intervals until the leaf discs were bleached. The Chls were successfully extracted from most species using this immersion method and yielded clear green solutions. However, this method was not applicable for several species, such as *Brassaiopsis glomerulata* and *Pteris vittata*. The leaf discs of these two species yielded a turbid brown solution when immersed in 80% acetone, indicating that the Chls were damaged due to unknown reasons. The total Chls and carotenoids (Cars) in the clear green extract were spectrophotometrically measured according to Wellburn^42^ and expressed on an area basis.

### Determination of the flavonoid content and antioxidant ability

The flavonoids and antioxidants were extracted from two leaf discs of each sample in 1.5 mL of methanol in a sealed centrifuge tube at 4 °C in the dark for 48 h. The flavonoid content in each extract was determined by aluminium chloride spectrophotometry^43^. Briefly, 0.5 mL of 10-fold diluted extract was mixed with 0.2 mL of 5% NaNO_2_, 0.3 mL of 10% AlCl_3_ (freshly prepared) and 1 mL of 1 M NaOH (freshly prepared). After standing for 5 minutes, the absorbance of the mixture was measured at 510 nm using another mixture made with deionized water instead of the flavonoid extract as a blank. The flavonoid content was calculated using a calibration curve constructed with 25–1000 μmol L^−1^ of catechin and expressed on an area basis.

The antioxidant ability in the extract was measured by a 1,1-diphenyl-2-picrylhydrazyl (DPPH) assay according to Nguyen *et al*.^44^ A 10 μL aliquot of the extract was added to 3 mL of 100 μM freshly prepared DPPH solution (in 95% methanol). After standing for 5 minutes, the absorbance of the mixture was measured at 517 nm using 95% methanol as a blank. The clearance rate of DPPH was calculated by a calibration curve made with 20–100 μM of DPPH. The antioxidant ability was expressed as μmol of DPPH scavenging capacity per unit area.

### Statistical analysis

All statistical procedures were performed using IBM SPSS Statistics v. 19.0 (IBM, NY, USA). A Student’s t test was used to examine the effects of season (winter, summer) on the anthocyanin, Chl and flavonoid contents and the antioxidant abilities based on conditional grouping of the species. Since the concentration-frequency distribution showed that the species with an anthocyanin content ranging from 0.02–0.04 μmol cm^−2^ occurred most frequently in both summer and winter, the first method for grouping the species was based on the condition that the anthocyanin content was >0.02 μmol cm^−2^. As a result, 29 species (*Castanopsis fissa, Craibiodendron kwangtungense, Smilax china, Sapium discolor, Desmos chinensis, Rubus alceaefolius, Rhaphiolepis indica, Microcos paniculata, Tetracera asiatica, Toxicodendron vernicifluum, Macaranga sampsonii, Sabia limoniacea, Engelhardtia, roxburghiana, Syzygium jambos, Rourea microphylla, Cryptocarya concinna, Cratoxylum cochinchinense, Alchornea trewioides, Bauhinia championi, Machilus pingii, Uncaria rhynchophylla, Syzygium rehderianum, Brassaiopsis glomerulata, Fissistigma glaucescens, Castanopsis chinensis, Marsdenia tinctoria, Aporosa dioica, Eriocarpous Glochidion*, and *Homalium cochinchinense*) were in the summer group (SI), and 14 species (*R. microphylla, R. indica, S. rehderianum, E. Glochidion, Ficus fistulosa, E. roxburghiana, C. kwangtungense, Calophyllum membranaceum, Eurya groffii, Blastus cochinchinensis, Ficus esquiroliana, S. jambos, A. dioica*, and *Acmena acuminatissima*) were in the winter group (WI). The second grouping method focused only on species that had red young leaves in both summer and winter, namely the intersection of the two seasons. Ten species (*B. cochinchinensis, S. rehderianum, C. kwangtungense, R. microphylla, E. roxburghiana, Garcinia oblongifolia, E. Glochidion, R. indica, A. dioica*, and *S. jambos*) met the criterion, and the corresponding summer and winter groups were designated SII and WII, respectively. The correlations between anthocyanins and the antioxidants and flavonoids in various species groups were analysed using Sigmaplot v. 12.5 (Systat Software Inc., USA).

## Supplementary information

Supplementary information
