# Supplementary material for: Seasonal variations in group leaf characteristics in species with red young leaves
Source: Sci Rep. 2019 Nov 11;9:16529. doi: 10.1038/s41598-019-52753-x (PMC6848096; doi:10.1038/s41598-019-52753-x)
Supplement: Supplementary file 1 — Supplementary information [file 41598_2019_52753_MOESM1_ESM.pdf]

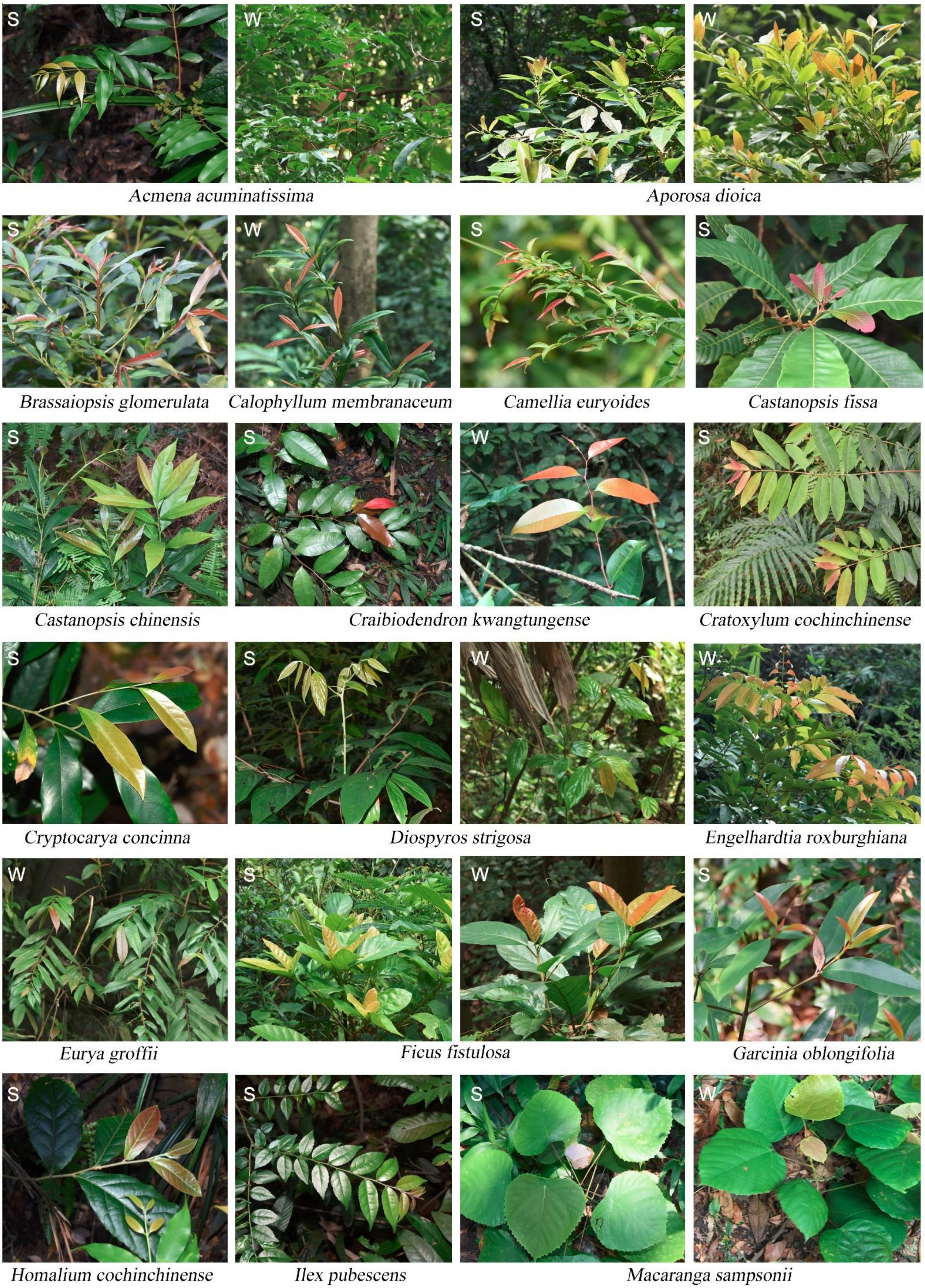

Figure S1. Species with red young leaves in the Dinghushan biosphere reserve (1/3). S=summer, W=winter. (Photo by T.-J. Zhang)

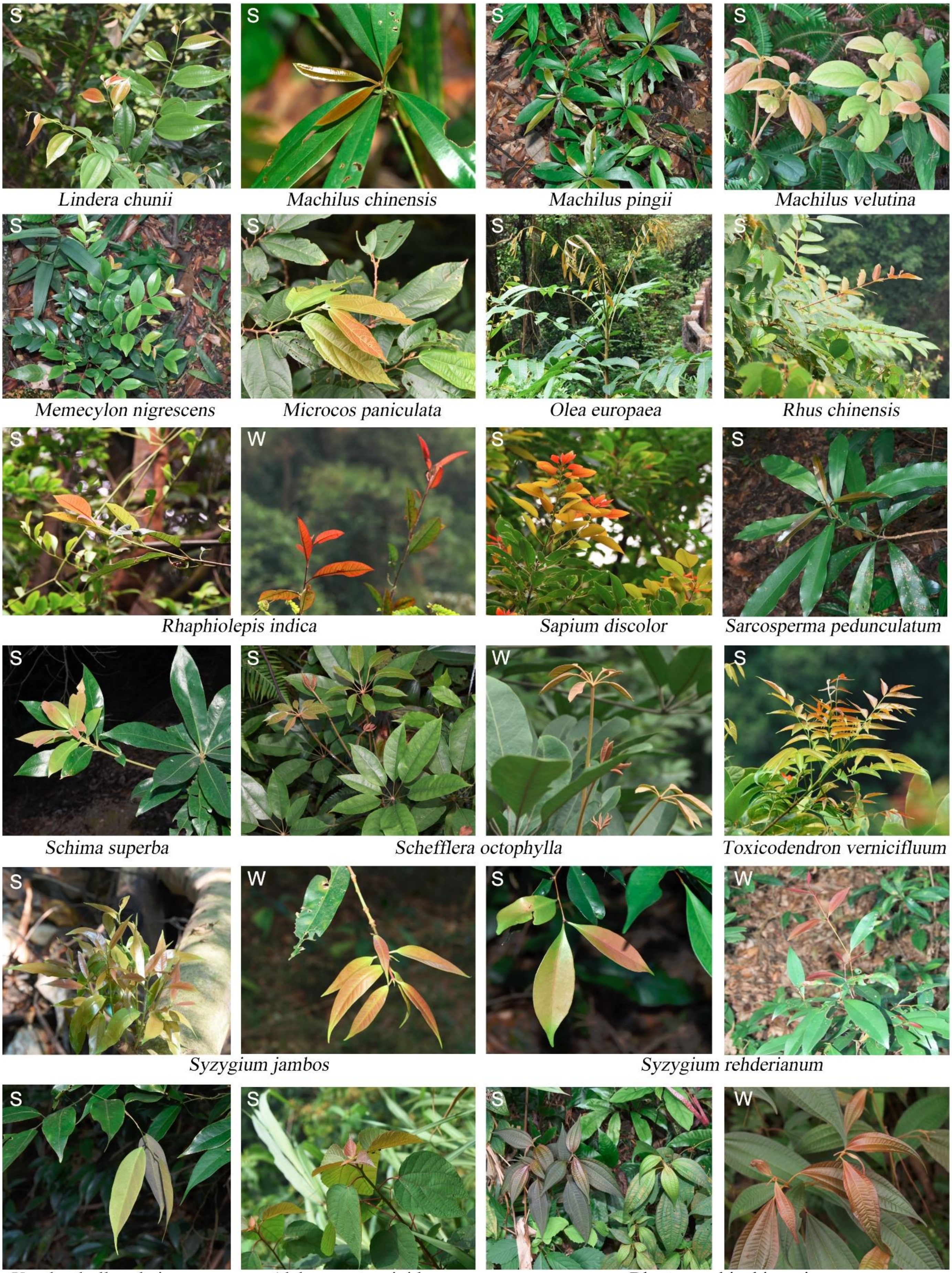

Figure S1. Species with red young leaves in the Dinghushan biosphere reserve (2/3). S=summer, W=winter. S=summer, W=winter. (Photo by T.-J. Zhang)

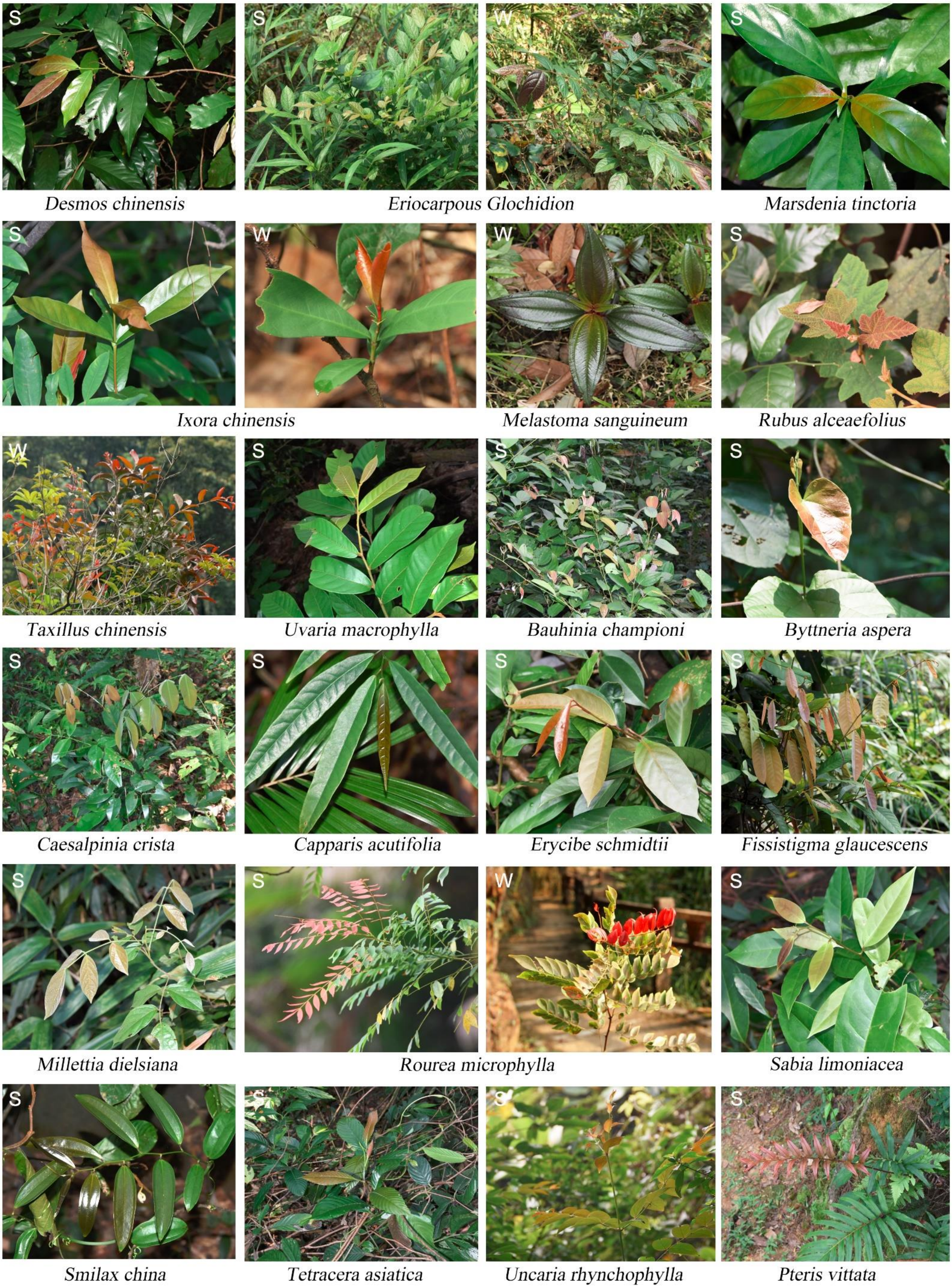

Figure S1. Species with red young leaves in the Dinghushan biosphere reserve (3/3). S=summer, W=winter. (Photo by T.-J. Zhang)
